# Ferroptosis in gouty arthritis: a potential therapeutic strategy

**DOI:** 10.3389/fimmu.2026.1769719

**Published:** 2026-02-05

**Authors:** Heguo Yan, Niqin Xiao, Bo Yang, Jian Zhang, Yundong Xu, Bingbing Chen, Sanjin Zeng, He Qian, Shengyi Zhao, Rong Wang, Zhaohu Xie, Zhaofu Li, Jing Xie

**Affiliations:** 1 Yunnan University of Chinese Medicine, Kunming, Yunnan, China; 2 Department of Rheumatology and Immunology, Zhaotong Hospital of Traditional Chinese Medicine, Zhaotong, Yunnan, China

**Keywords:** cell death, ferroptosis, gouty arthritis, inflammatory response, targeted therapy

## Abstract

Ferroptosis, an emerging form of iron-dependent programmed cell death, has recently gained substantial research interest due to its involvement in various inflammatory disorders. Gouty arthritis (GA), a chronic inflammatory disease driven by the deposition of monosodium urate crystals, is characterized by a complex interplay between cell death pathways and inflammatory responses. Despite growing evidence linking ferroptosis to inflammatory regulation, its precise contribution to the onset and progression of GA remains unclear. This systematic review synthesizes current molecular insights into ferroptosis and examines its potential regulatory role in GA pathophysiology. By integrating recent experimental and clinical advances, it evaluates the therapeutic promise of targeting ferroptosis in GA. Through comprehensive analysis of ferroptosis-associated signaling networks and GA-related pathological events, this review aims to provide a stronger mechanistic foundation and highlight future directions for disease research and targeted therapeutic development.

## 1 Introduction

Gouty arthritis (GA) is a form of inflammatory arthritis caused by abnormalities in purine metabolism and/or impaired renal excretion of uric acid, resulting in persistent hyperuricemia and deposition of monosodium urate (MSU) crystals in joint spaces and periarticular tissues. GA is classified as a metabolic rheumatic disorder (1–3). Epidemiological data show a global prevalence of approximately 1–4% and an incidence of 0.1–0.3%, with a male-to-female ratio ranging from 3:1 to 10:1; notably, its prevalence continues to rise worldwide (4, 5). Notably, driven by multiple factors—including the accelerating pace of global population aging, the widespread adoption of high-purine (purine-rich), high-fat diets, and the rising prevalence of metabolic syndrome—the overall incidence of gout continues to rise; it has thus become a significant metabolic disease burden that cannot be ignored in the global public health field (6). As the disease progresses with recurrent flares, patients often develop comorbidities such as diabetes, chronic kidney disease, and cardiovascular complications. Accumulated MSU crystals can further induce joint destruction, bone erosion, deformity, and long-term disability, markedly diminishing quality of life (7). Current therapeutic strategies primarily aim to reduce serum uric acid levels and suppress inflammation, utilizing agents such as colchicine, nonsteroidal anti-inflammatory drugs (NSAIDs), and glucocorticoids. However, these treatments are constrained by limited efficacy, frequent relapses, and notable adverse effects, underscoring the need to investigate novel pathogenic mechanisms and develop more effective therapeutic approaches (8, 9). Specifically, in clinical practice, existing medications often prove ineffective for some patients with refractory gouty arthritis, failing to adequately control joint inflammation flare-ups and disease progression. For GA patients with concomitant chronic kidney disease, drugs like nonsteroidal anti-inflammatory drugs (NSAIDs) carry clear contraindications, while medications such as colchicine require strict dose adjustments, significantly limiting treatment options. Additionally, elderly GA patients, due to declining physiological functions and frequent comorbidities, generally exhibit poor tolerance to existing medications. They are prone to adverse reactions such as gastrointestinal disturbances and hepatic/renal impairment, further exacerbating treatment challenges (10).

In recent years, ferroptosis has emerged as a novel form of programmed cell death and has attracted substantial attention for its distinct biological characteristics. Defined as an iron-dependent process driven by excessive lipid peroxidation, ferroptosis differs fundamentally from classical cell death modalities such as apoptosis, necrosis, and pyroptosis. Its key molecular features include dysregulated iron metabolism, inactivation of glutathione peroxidase 4 (GPX4), and accumulation of lipid peroxide, ultimately causing oxidative damage to cellular membranes and cell death (11–13). Morphologically, ferroptotic cells exhibit shrunken mitochondria, increased membrane density, reduced or absent cristae, and outer mitochondrial membrane rupture, while preserving overall plasma membrane integrity, normal nuclear size, and absence of chromatin condensation. Immunologically, ferroptosis is often accompanied by pronounced pro-inflammatory responses (14–17). Increasing evidence indicates that ferroptosis not only participates in various pathological processes such as tumors, neurodegenerative diseases, and cardiovascular diseases (18–20), but is also closely associated with multiple inflammatory and autoimmune diseases including osteoarthritis, rheumatoid arthritis, and systemic lupus erythematosus (21–24). Notably, a study has demonstrated a positive correlation between systemic iron levels and the frequency of GA flares (25). Experimental findings further reveal that iron supplementation aggravates synovial inflammation in rat models of arthritis (26), while iron chelation therapy reduces inflammatory responses by lowering iron ion levels (27). These observations collectively suggest that ferroptosis may be intimately involved in GA pathogenesis. Despite these associations, the precise mechanisms linking ferroptosis to GA remain insufficiently understood. This review summarizes the molecular regulatory networks governing ferroptosis and explores its potential pathological role in GA. By providing deeper insight into the underlying disease mechanisms, it offers a theoretical basis for the development of ferroptosis-targeted therapeutic strategies, which may hold significant clinical potential for GA management (28–30). Therefore, ferroptosis represents a promising emerging target for future GA therapy.

## 2 Molecular mechanisms of ferroptosis and its biological characteristics

### 2.1 Sources, functions, storage, and conversion forms of iron

Iron is an essential trace element required for numerous physiological processes in the human body, with a total content of approximately 4–5 grams in healthy adults. It plays a key role in cellular proliferation and growth, particularly in erythrocyte maturation and functions as a vital cofactor for various metabolic enzymes involved in oxygen transport, DNA synthesis, and energy metabolism (31–34). The human body ingests and absorbs 1–2 mg of iron daily through diet to maintain homeostasis. Dietary iron is present primarily in the forms of Fe²^+^ and Fe³^+^, and its metabolism involves four major steps: absorption, transport, storage, and utilization. The central regulatory mechanism ensures precise control of iron uptake, release from storage sites, mobilization for metabolic needs, and limited excretion, thereby maintaining stable intracellular iron levels and preventing iron overload, which can otherwise trigger oxidative stress and cellular damage (35). Dietary iron exists in two main forms: heme iron derived from animal-based foods and non-heme iron obtained from plant sources. Absorption occurs predominantly in the duodenum and upper jejunum and is tightly regulated by key iron transporters such as divalent metal transporter 1 (DMT1) and ferroportin 1 (FPN1). Dietary Fe³^+^ is initially reduced to Fe²^+^, after which it is taken up into intestinal epithelial cells via DMT1. A fraction of this absorbed iron is exported into the bloodstream through FPN1, where it binds to transferrin and is subsequently delivered to tissues and organs with iron requirements (36). Another fraction is stored intracellularly in the form of ferritin, particularly within macrophages in the liver, spleen, and bone marrow (37, 38). Under conditions of iron deficiency or increased physiological demand, ferritin-bound Fe³^+^ is mobilized through ferritinophagy, reduced again to Fe²^+^, and made available for metabolic use. Both iron deficiency and iron overload can significantly disrupt normal physiological functions and contribute to disease development (39, 40).

### 2.2 Iron metabolism dysregulation as the core driver of ferroptosis

**Figure 1:**
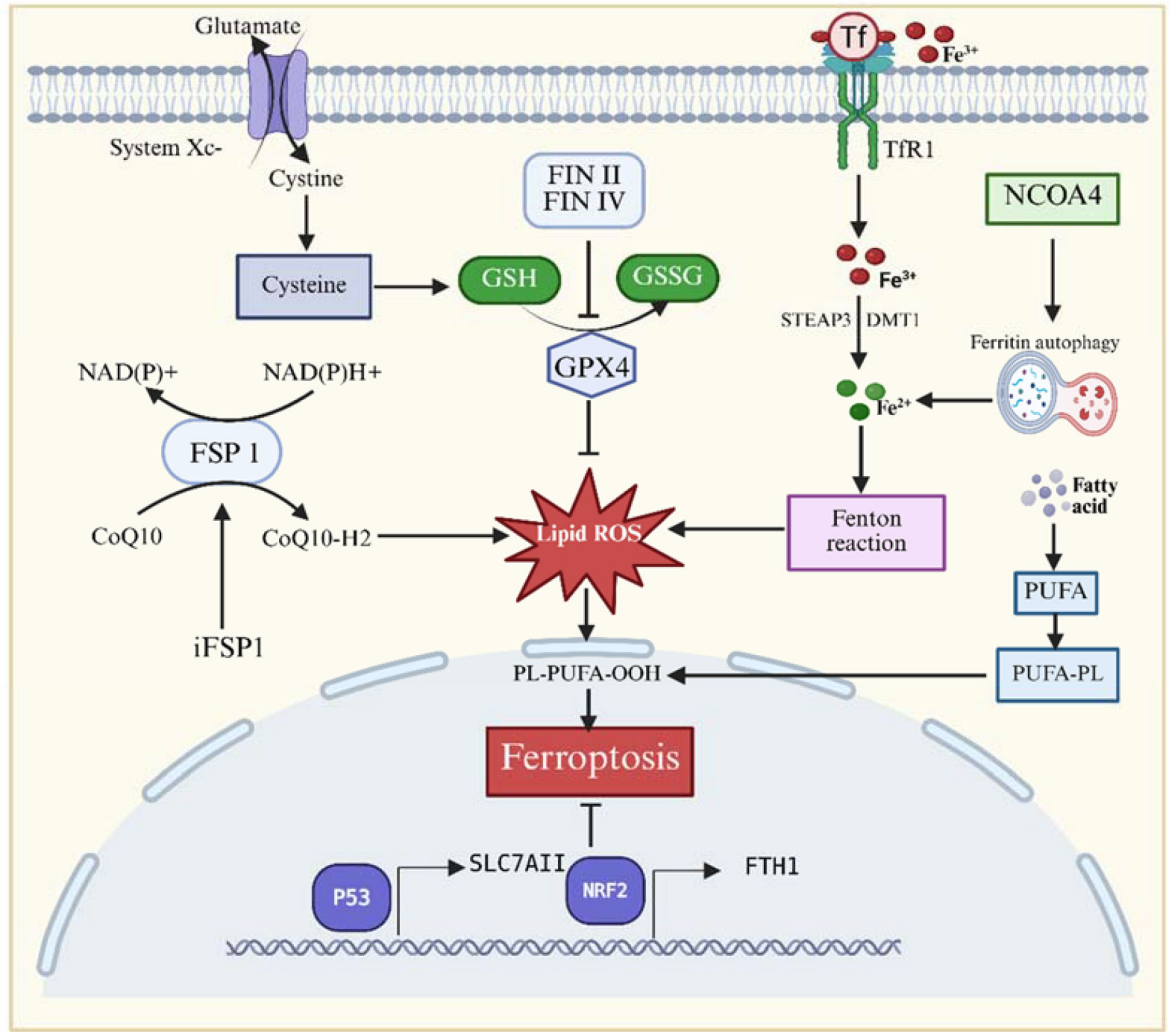
Schematic Overview of the Molecular mechanisms underlying ferroptosis. System Xc^−^ facilitates cystine import, supporting glutathione (GSH) synthesis, which in turn enables glutathione peroxidase 4 (GPX4) to detoxify lipid reactive oxygen species (ROS). In parallel, the FSP1–coenzyme Q10 (CoQ10) axis provides an additional defense by directly scavenging lipid radicals. Iron homeostasis also plays a central role: transferrin (Tf)–transferrin receptor 1 (TfR1) signaling promotes cellular iron uptake, while iron overload enhances oxidative stress through Fenton reactions and NCOA4-mediated ferritinophagy. Polyunsaturated fatty acids (PUFAs) are esterified into phospholipids (PUFA-PL), which undergo oxidation to phospholipid hydroperoxides (PL-PUFA-OOH), serving as key executors of ferroptosis. Transcriptional regulators modulate these processes, with p53 suppressing SLC7A11 to reduce antioxidant capacity, whereas NRF2 protects against ferroptosis by regulating iron metabolism and GSH synthesis. The combined effects of GPX4 inhibition, excessive iron accumulation, and cysteine depletion converge to amplify lipid peroxidation, ultimately driving ferroptotic cell death.

Ferroptosis is a distinct form of programmed cell death characterized by the intracellular accumulation of iron ions. Its central mechanism involves iron-driven lipid peroxidation, whereby iron ions participate in Fenton chemistry to generate excessive reactive oxygen species (ROS). These ROS trigger oxidative damage to cellular membranes, disrupt membrane integrity, and impair cellular function, ultimately resulting in cell death (41–43), (**Figure 1**). The initiation of ferroptosis requires disruption of iron homeostasis, which is maintained through coordinated regulation of iron uptake, storage, and export. Transferrin receptor 1 (TfR1) is responsible for importing extracellular iron into cells, ferritin acts as the main intracellular iron reservoir, and ferroportin (FPN), the only known iron exporter regulated by hepcidin, controls iron efflux. Disturbances in any of these processes can result in iron overload, forming a key driving force for ferroptosis (44, 45). Abnormal iron uptake is primarily characterized by excessive expression of transferrin receptor 1 (TfR1). Pathological conditions such as inflammation and hypoxia activate transcription factors including HIF-1α and NF-κB, which upregulate TfR1 and thereby increase endocytosis of Fe³^+^. After endocytosis, Fe³^+^ is reduced to Fe²^+^ and transported into the labile iron pool (LIP) via divalent metal transporter 1 (DMT1). The elevated Fe²^+^ pool provides substrates for Fenton chemistry and enhances lipoxygenase (LOX)-mediated oxidation of polyunsaturated fatty acids (PUFAs) to generate lipid hydroperoxides (LOOH) (46–48). Dysregulated iron storage further contributes to intracellular Fe²^+^ accumulation through enhanced release and reduced synthesis of ferritin. The autophagy receptor NCOA4 promotes ferritin degradation via ferritinophagy, liberating Fe²^+^ into the cytosol. Concurrently, oxidative stress inactivates iron regulatory proteins (IRPs) and damages ribosomal function, collectively impairing ferritin synthesis and intensifying iron overload (49–51). Impaired iron export also aggravates pathological iron retention. Chronic inflammatory states, activation of the BMP–SMAD signaling pathway, or mutations in FPN can cause excessive hepcidin production or increased FPN binding to ferritin. These events accelerate FPN internalization and degradation, thereby limiting iron efflux (52). These three factors synergistically form an “iron accumulation-oxidative stress” positive feedback loop. ROS further suppress FPN function, promoting the conversion of LOOH into toxic lipid aldehydes that disrupt cell membrane integrity, ultimately triggering ferroptosis (53).

### 2.3 Role of lipid peroxidation in ferroptosis

Lipid peroxidation is a central execution step in ferroptosis and is regulated through multiple interconnected mechanisms that directly determine cell fate. Polyunsaturated fatty acids (PUFAs) incorporated into membrane phospholipids provide abundant substrates for oxidative reactions. When intracellular free iron catalyzes the generation of reactive oxygen species (ROS) through the Fenton reaction, a chain reaction of PUFA lipid peroxidation is initiated. The primary oxidation products, such as HpODEs and HpETEs, disrupt membrane organization and increase permeability, ultimately compromising membrane integrity (54–58). Two major pathways drive lipid peroxidation: enzymatic and non-enzymatic. In the enzymatic pathway, lipoxygenases (LOXs) catalyze the oxidation of PUFAs to generate specific hydroperoxide isomers such as 13-HpODE, and LOX activation significantly enhances susceptibility to ferroptosis. The non-enzymatic pathway relies on free radical–mediated reactions, including hydrogen atom transfer (HAT) and peroxyl radical addition (PRA), producing diverse products such as truncated lipids and electrophilic species that further damage membranes (59, 60). Cells counteract lipid peroxidation through the glutathione (GSH)–glutathione peroxidase 4 (GPX4) system. GPX4 uses GSH as a reducing agent to convert lipid hydroperoxides (L-OOH) into their corresponding hydroxyl derivatives (L-OH), thereby neutralizing oxidative reactivity. When GPX4 is inhibited or GSH levels are depleted, lipid peroxides accumulate unchecked, leading to ferroptotic cell death (54). In addition to the GPX4-dependent antioxidant pathway, the endoplasmic reticulum–associated protein TXNDC12 provides an alternative protective mechanism against lipid peroxidation. Loss of TXNDC12 abolishes this protective effect and significantly increases cellular vulnerability to ferroptosis (61–63). As lipid peroxidation progresses, its secondary products, such as 4-hydroxy-2-nonenal and malondialdehyde, intensify cell death signaling by forming covalent cross-links with proteins and nucleic acids. These modifications impair mitochondrial and endoplasmic reticulum functions and activate stress-responsive pathways, including NRF2 and ATF4 signaling (64, 65). Overall, ferroptosis is driven by the combined effects of iron-dependent radical reactions, enzyme-mediated oxidation, and failure of antioxidant defense systems, culminating in overwhelming lipid peroxidation and cell death.

### 2.4 Ferroptosis-related signaling pathways and regulatory factors

System Xc^−^, a glutamate–cystine antiporter, is essential for GSH synthesis. By facilitating the uptake of extracellular cystine, it sustains intracellular GSH levels and supports the antioxidant activity of GPX4. Inhibition of System Xc^−^ reduces intracellular cystine availability, decreases GSH synthesis, compromises GPX4 function, and ultimately promotes lipid peroxidation and ferroptosis (66). Transcription factors p53 and Nrf2 play pivotal yet distinct regulatory roles in ferroptosis. p53 can promote ferroptosis by repressing the expression of solute carrier family 7 member 11 (SLC7A11), thereby reducing System Xc^−^ activity; at the same time, p53 also modulates additional antioxidant genes in a manner that can suppress ferroptosis, reflecting its context-dependent duality (67, 68). Nrf2, a master regulator of cellular antioxidant responses, activates the transcription of numerous protective genes, including SLC7A11, GPX4, and several iron-regulating proteins, thereby effectively inhibiting ferroptosis progression (69). Beyond these classical pathways, newly identified regulatory mechanisms have further expanded the understanding of ferroptosis. Ferroptosis suppressor protein 1 (FSP1) reduces coenzyme Q10 (CoQ10) to its active antioxidant form, ubiquinol, which functions as a potent lipid radical scavenger and inhibits ferroptosis independently of GPX4 (70, 71). Collectively, the ferroptosis regulatory network integrates metabolic control, transcriptional regulation, and antioxidant defense, providing a comprehensive framework for its exploration as a promising therapeutic target.

## 3 Pathogenesis of GA and its relationship with ferroptosis

### 3.1 Mechanism of urate crystal-induced inflammatory response in GA

**Figure 2:**
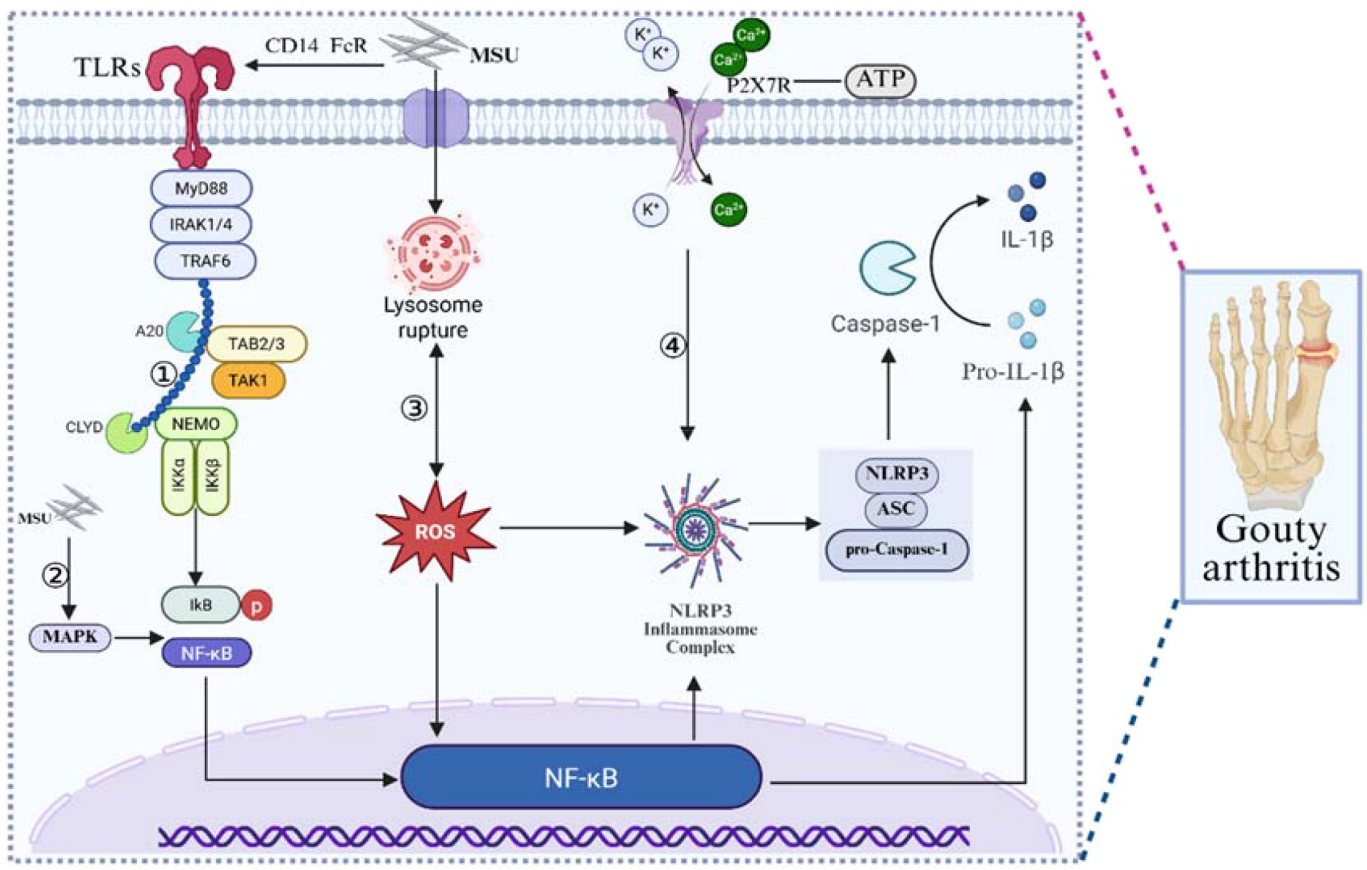
Pathogenic mechanisms underlying MSU crystal–driven inflammation in GA. ①MSU crystals aberrantly activate the MAPK signaling pathway, leading to ubiquitination and degradation of IκBα. This allows NF-κB to translocate into the nucleus, where it binds κB motifs on target gene promoters and drives transcription of multiple inflammatory mediators, including the precursors pro-IL-1β and pro-IL-18. ②MSU indirectly activates Toll-like receptors (TLRs) by interacting with surface molecules such as Fc receptors and CD14. Activated TLRs signal through the adaptor protein MYD88, further stimulating NF-κB and enhancing the transcription and synthesis of pro-inflammatory cytokines. ③ MSU induces lysosomal rupture and ROS production, which reinforce NF-κB activation. NF-κB promotes transcription of NLRP3, ASC, and pro-Caspase-1, enabling assembly of the NLRP3 inflammasome. Inflammasome activation drives Caspase-1–mediated maturation and secretion of IL-1β. ④MSU increases extracellular ATP levels, which activate P2X7 receptors and trigger substantial K^+^ efflux and Ca²^+^ influx. These ionic changes accelerate NLRP3 inflammasome activation, enhance Caspase-1 production, and further promote IL-1β maturation and release.

The fundamental pathogenesis of GA arises from disturbances in uric acid metabolism that lead to the formation and deposition of MSU crystals within joints and periarticular tissues. These crystals activate the innate immune system and initiate robust inflammatory responses. Among the key mediators involved, NF-κB and NLRP3 play central roles in driving GA-associated inflammation. MSU crystals aberrantly activate the MAPK signaling cascade, promoting ubiquitination and subsequent degradation of IκBα. This process releases NF-κB from its inhibitory complex, allowing it to translocate into the nucleus, bind κB motifs on target gene promoters, and induce transcription of pro-inflammatory cytokines such as pro-IL-1β and pro-IL-18, along with other inflammatory mediators including TNF-α (72). Concurrently, MSU interacts with cell surface proteins like Fc receptors and CD14 to activate Toll-like receptors (TLRs). TLR activation signals through the adaptor protein MyD88, leading to further activation of NF-κB and amplification of downstream inflammatory gene expression, thereby contributing to the initiation or worsening of GA inflammatory episodes (73). NLRP3 also plays a pivotal role by directly sensing MSU crystals in tissues. Upon activation, NLRP3 oligomerizes in an ATP-dependent manner, after which the adaptor protein ASC recruits pro-Caspase-1 to assemble the NLRP3 inflammasome complex (74). This inflammasome subsequently drives the maturation and secretion of IL-1β and IL-18, reinforcing the inflammatory cascade characteristic of GA. Within the assembled inflammasome complex, pro-Caspase-1 undergoes autocatalytic cleavage to form the active heterodimeric enzyme Caspase-1. Activated Caspase-1 then processes pro-IL-1β and pro-IL-18 into their mature, bioactive forms, thereby initiating potent inflammatory responses. In addition to cytokine maturation, Caspase-1 also cleaves Gasdermin D, generating its pore-forming N-terminal fragment. This fragment disrupts the plasma membrane, promotes pyroptotic cell death, and further amplifies inflammation in GA (9). During the early stages of acute gouty arthritis (AGA), necrotic and infiltrating inflammatory cells release high levels of extracellular ATP through membrane channels or connexins. Extracellular ATP binds to P2X7 receptors (P2X7R) on the cell surface. As an upstream regulator of NLRP3, P2X7R can be activated directly or indirectly by MSU crystals. Upon activation, P2X7R forms transient membrane pores that facilitate Ca²^+^ influx and K^+^ efflux, both of which are key signals for NLRP3 inflammasome activation. This promotes the maturation and secretion of IL-1β and IL-18, thereby initiating or exacerbating inflammatory flares in AGA (75–78), (**Figure 2**).

### 3.2 Iron influences purine metabolism and is a risk factor for hyperuricemia and gout formation

GA is an inflammatory joint disease that develops secondary to hyperuricemia (79). Although hyperuricemia and gout are often regarded as lifestyle-related conditions linked to prolonged intake of high-purine, animal-derived foods, growing evidence suggests that the iron content in these foods may also contribute to disease onset (80, 81). Total body iron is determined largely by dietary intake, and foods rich in heme iron, including seafood, red meat, and organ meats, represent major contributors to iron overload, especially in men (82). Recent research has shown that disturbances in iron metabolism are closely involved in the development of hyperuricemia and gout, with dietary iron from high-purine foods identified as a potential initiating factor (83–86). A cohort study reported a strong positive association between serum ferritin concentrations and the prevalence of hyperuricemia, as well as a close correlation with serum uric acid levels (25). Consistent with these findings, a large national study in China demonstrated that serum ferritin, transferrin, and soluble transferrin receptor levels are all positively associated with the risk of hyperuricemia (87). Further analyses of serum iron parameters, including ferritin, transferrin saturation, and transferrin, revealed that hereditary iron overload is linked to an increased incidence of gout. Furthermore, iron overload significantly increases the risk of hyperuricemia and gout attacks by elevating serum uric acid levels (88, 89).

### 3.3 Iron metabolism abnormalities play a significant role in GA

Iron metabolism dysregulation plays an important role in the development of GA. Its pathogenic effects mainly involve amplification of inflammatory responses, promotion of joint tissue injury, and modulation of uric acid crystal–associated processes, thereby offering insight into potential therapeutic targets. Clinical observations show that patients with gout have significantly elevated iron concentrations in synovial fluid and periarticular tissues, indicating that iron overload may intensify inflammation and contribute to structural damage (90). Supporting this, Shen et al. (91) demonstrated in an AGA mouse model that intravenously administered dextran iron induced marked iron accumulation and led to a substantial reduction in both mechanical and thermal pain thresholds compared with control animals, confirming that iron excess worsens AGA symptoms. Mechanistically, elevated serum or tissue iron increases the production of ROS through the Fenton reaction, which activates the NLRP3 inflammasome, a central mediator of GA-related inflammation. ROS also enhances the responsiveness of monocytes and macrophages to MSU crystals in the presence of iron ions, resulting in augmented IL-1β secretion. Similar mechanisms have been described in studies of autoimmune hepatitis and COVID-19, indicating a broader relevance of iron-driven inflammasome activation (92–94). In the context of joint tissue injury, iron overload induces ferroptosis, a lipid peroxidation-dependent cell death pathway occurring in synovial cells and chondrocytes. This leads to the release of DAMPs, recruitment of neutrophils, and strengthening of the inflammatory cascade, a process also observed in research on diabetic osteoporosis and neuroinflammation (95, 96). In relation to uric acid crystal regulation, iron ions have a significant impact on xanthine oxidase (XO) activity. Excess iron can enhance uric acid synthesis and reduce its solubility, thereby accelerating the formation of MSU crystals. Additionally, iron retention within macrophages, partly driven by hepcidin upregulation and restricted iron export, impairs their ability to phagocytose and clear MSU crystals, ultimately prolonging the inflammatory response (94, 97). As central regulators of iron homeostasis, macrophages are particularly vulnerable to iron overload–induced ferroptosis, which further aggravates local inflammation. Under conditions of elevated iron, increased uptake via TfR1 and DMT1, together with ferritinophagy-mediated ferritin degradation, leads to intracellular iron accumulation. This accumulation predisposes macrophages to ferroptotic cell death, resulting in the release of DAMPs such as HMGB1 and ATP. These DAMPs recruit neutrophils and stimulate the secretion of proinflammatory mediators including IL-6 and TNF-α. These cytokines can additionally upregulate TfR1 expression in parenchymal cells, such as hepatocytes and renal tubular epithelial cells, promoting further iron uptake. Concurrent activation of the NF-κB pathway and suppression of FPN expression reduce iron efflux, establishing an “inflammation–iron dysregulation–ferroptosis” cycle that amplifies tissue (98, 99). Based on these mechanistic insights and supporting evidence from other inflammatory disorders, therapeutic strategies such as iron chelators (for example, deferoxamine) and ferroptosis inhibitors (for example, ferrostatin-1) may help mitigate inflammatory damage by limiting ferroptosis. Moreover, targeting hepcidin regulation or activating heme oxygenase-1 (HO-1) through agents such as melatonin to restore iron balance may effectively alleviate inflammation in GA. These approaches present promising therapeutic directions for future intervention.

### 3.4 Potential mechanisms of ferroptosis in GA

#### 3.4.1 Ferroptosis-induced GA pathogenesis

Ferroptosis has emerged as an important contributor to the development of inflammatory diseases. Both processes share core features such as iron-dependent oxidative stress and lipid peroxidation, and activation of inflammatory signaling pathways can precipitate ferroptosis, whose immunological outcomes often present as pro-inflammatory responses (100). As a key component of oxidative stress, ferroptosis is inherently linked to the pathophysiology of GA. Elevated serum uric acid and the deposition of MSU crystals within the joint cavity activate innate immune mechanisms, leading to the synthesis and release of inflammatory cytokines, which are central drivers of GA onset (101). Clinical evidence further supports the involvement of ferroptosis in GA. In a study involving 12 gout patients, iron chelation therapy that reduced systemic iron stores to the minimal level required for normal erythropoiesis (without causing anemia) resulted in a marked reduction in both the frequency and severity of gout flares during the maintenance phase. The therapeutic effect was hypothesized to result from bloodletting-induced decreases in circulating iron, enhanced efflux of intracellular Fe²^+^, alleviation of intracellular Fe²^+^ overload, and subsequent suppression of ferroptosis, ultimately mitigating joint inflammation (102). Experimental findings in animal models provide additional confirmation. In GA mice, joint tissues displayed significantly increased levels of free iron, nitric oxide (NO), and malondialdehyde (MDA), a key marker of ferroptosis, along with a pronounced decline in GSH levels (103). Concurrently, clinical studies revealed abnormally elevated serum ferritin and transferrin levels in GA patients, positively correlated with GA attack frequency (25). Moreover, GA joint tissues consistently exhibit high MDA concentrations and reduced GSH content, reflecting a sustained state of oxidative stress. Collectively, these clinical and preclinical observations indicate that ferroptosis likely plays an important mechanistic role in the development and progression of GA.

#### 3.4.2 Ferroptosis promotes GA progression: tophi formation and bone-joint destruction

Tophi, the characteristic granulomatous structures of advanced gout, consist of a central core of MSU crystals surrounded by chronic inflammatory cells and fibrotic tissue. Emerging evidence suggests that ferroptosis may contribute substantially to tophus formation and progression through several interconnected mechanisms. Iron ions influence the pathogenesis of tophi by modulating XO activity. Elevated iron levels enhance XO-mediated uric acid synthesis while decreasing uric acid solubility, thereby facilitating MSU crystal nucleation and growth. Concurrently, hepcidin-induced iron retention within macrophages diminishes their ability to phagocytose and clear urate crystals, promoting persistent crystal accumulation (94, 97). Under the chronic inflammatory conditions characteristic of GA, synovial and tissue macrophages repeatedly exposed to MSU crystals become increasingly susceptible to ferroptosis. Ferroptotic macrophages exhibit severely impaired phagocytic and clearance function, allowing MSU crystals to accumulate and form the initial “crystal nucleus” around which tophi subsequently develop (104). The release of DAMPs such as HMGB1 during ferroptosis activates macrophages and persistently activates the NLRP3 inflammasome. This results in enhanced IL-1β secretion, amplifying necrotic inflammation and providing a critical stimulus for granuloma formation (95). In addition, lipid peroxidation products, which are central biochemical features of ferroptosis, modify the local microenvironment in a manner that favors heterogeneous nucleation and aggregation of MSU crystals. These oxidative lipid byproducts can also impair fibroblast function, disrupt extracellular matrix metabolism, and hinder proper fibrotic encapsulation of tophi, ultimately destabilizing lesion architecture (92). More importantly, the DAMPs released from ferroptotic cells markedly enhance the ability of MSU crystals to stimulate neutrophils. This amplification increases NLRP3 inflammasome activation and promotes neutrophil extracellular trap formation (NETosis), resulting in the generation of aggregated neutrophil extracellular traps (aggNETs). MSU crystals that become enclosed within these aggNET structures exhibit strong resistance to degradation by deoxyribonuclease I (DNase I) and evade effective clearance, which leads to their persistent retention within joint tissues and contributes to the long-term deposition that ultimately forms tophi (105–107). Taken together, these findings indicate that ferroptosis may represent a central mechanistic driver of tophus development by sequentially impairing immune-mediated crystal clearance, intensifying inflammatory responses, modifying the local microenvironment, and disrupting NET function.

Long-standing, recurrent gout attacks can progress to chronic gouty arthritis, which is characterized by bone erosion, focal cartilage degradation, cartilage defects, and aberrant new bone formation, ultimately culminating in irreversible joint destruction (108). Within this chronic inflammatory microenvironment, ferroptosis contributes to synovial and chondrocyte dysfunction by disrupting cellular redox balance and lipid metabolism. Sustained accumulation of ROS activates abnormal intracellular signaling pathways and impairs the cell’s ability to repair metabolic injury, thereby driving persistent inflammatory responses. At the same time, iron overload perturbs intracellular iron homeostasis, accelerates lipid peroxidation, and induces regulated cell death, establishing a self-perpetuating cycle that exacerbates joint tissue destruction (109). Studies provide further mechanistic insights into this process. Jing et al. (110) reported that IL-1β or TNF-α stimulation increases the expression of iron influx transporters such as TfR1 and DMT1 in chondrocytes, while reducing the expression of iron efflux transporters, thereby predisposing cells to intracellular iron accumulation. In a mouse model generated by intravenous injection of dextran iron, pronounced chondrocyte degeneration, subchondral bone destruction, and sclerosis were observed, accompanied by elevated expression of catabolic enzymes including ADAMTS5 and MMP13 and an increased number of osteoclasts. Reducing DMT1 expression diminished iron uptake, and pharmacologic inhibition of MAPK and PI3K/AKT/NF-κB signaling attenuated IL-1β-induced inflammation and extracellular matrix degradation in chondrocytes. Yao et al. (111) further demonstrated that both IL-1β-induced inflammation and dextran iron-induced iron overload in chondrocytes lead to substantial accumulation of intracellular and lipid ROS, along with increased expression of GPX4, SLC7A11, ACSL4, and p53, collectively promoting ferroptotic cell death. Treatment with ferrostatin-1 effectively inhibited this process. Taken together, these findings support the notion that ferroptosis contributes to the progression of gouty arthritis and plays a significant role in driving chronic joint destruction.

#### 3.4.3 The FPN1/ROS pathway may promote GA by activating acid-sensing ion channel 1α through ferroptosis

Iron metabolism–related proteins are centrally involved in the pathogenesis of GA through their regulatory effects on ferroptosis and inflammatory signaling. Among these proteins, ferroportin 1 (FPN1) is essential for maintaining intracellular iron balance. When FPN1 function is impaired, Fe²^+^ accumulates within cells, promoting the Fenton reaction and generating excessive ROS. This oxidative burden induces ferroptosis and triggers downstream inflammatory reactions (112). ASIC1α is another key mediator implicated in inflammatory joint disorders. During GA, phagocytosis of MSU crystals and subsequent intracellular ROS accumulation lead to lysosomal swelling and rupture, releasing acidic contents (pH 3.5–5.5) into the cytosol. After ferroptotic cell death, these acidic components are also released into the extracellular space, contributing to local acidification within the joint. Such acidification activates ASIC1α, resulting in increased extracellular Ca²^+^ influx and intracellular Ca²^+^ overload. This calcium dysregulation enhances NF-κB nuclear translocation and transcriptional activity and stimulates NLRP3 inflammasome activation. Consequently, the production and secretion of inflammatory cytokines, including IL-1β, IL-8, and TNF-α, are markedly elevated, aggravating joint inflammation (98, 113, 114). Based on these mechanistic insights, we propose that ferroptosis driven by the FPN1–ROS pathway may contribute to GA progression through the activation of ASIC1α.

#### 3.4.4 ASIC1α may exacerbate GA severity by regulating nuclear receptor coactivator 4 and ferritin heavy chain 1 pathway-mediated ferroptosis

**Figure 3:**
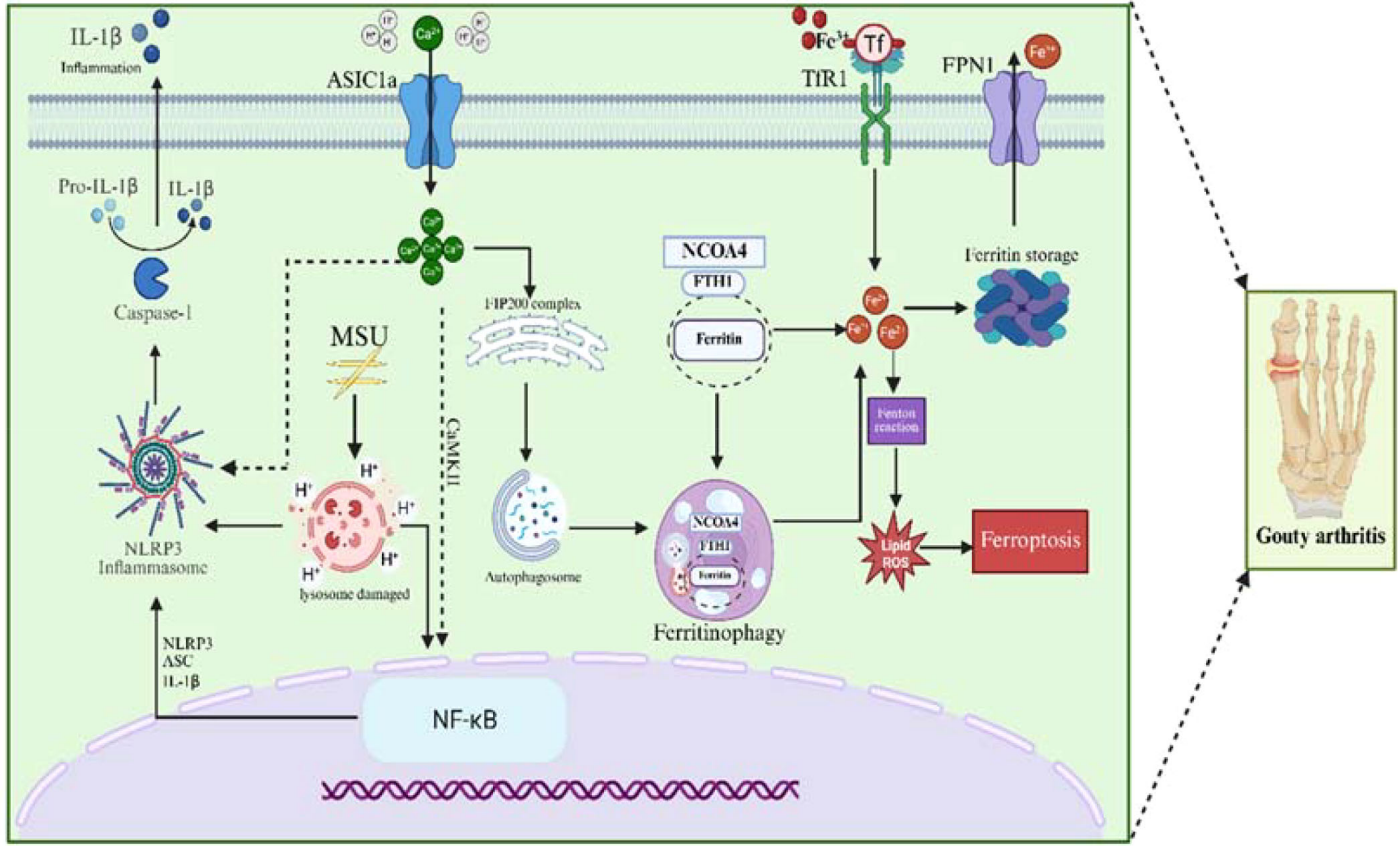
Molecular mechanisms by which ferroptosis contributes to GA progression. MSU crystal–induced injury disrupts lysosomal integrity, resulting in the release of H^+^ ions into the cytoplasm and subsequent activation of the NLRP3 inflammasome. This activation promotes the Caspase-1–mediated cleavage of pro-IL-1β into mature IL-1β, initiating the inflammatory response. At the same time, inflammation-associated signals stimulate the NF-κB pathway, which plays a key role in downstream transcriptional regulation. Within this inflammatory microenvironment, ASIC1α becomes activated, contributing to local tissue acidification. The acidic milieu further enhances ASIC1α activation, leading to increased Ca²^+^ influx. The resulting intracellular Ca²^+^ overload facilitates NF-κB nuclear translocation and transcriptional activation, as well as NLRP3 inflammasome stimulation. These events collectively promote the synthesis and extracellular release of inflammatory cytokines, thereby sustaining or intensifying joint inflammation. H^+^-mediated activation of ASIC1α induces Ca²^+^ influx, and elevated intracellular Ca²^+^ triggers calcium transients at the endoplasmic reticulum surface, promoting autophagosome formation and facilitating cellular ferroptosis. The progression of ferroptosis is accompanied by accumulation of free Fe²^+^, which further accelerates the advancement of gouty arthritis. In parallel, iron stored in ferritin can be exported through FPN1, which contributes to maintaining intracellular iron homeostasis.

Ferroptosis is closely linked to the maintenance of physiological cellular iron homeostasis. As an autophagy-dependent form of regulated cell death, ferroptosis relies on upstream autophagic processes that modulate the ferroptotic cascade (115). A central component of this regulation is NCOA4, which mediates ferritinophagy, the selective autophagic degradation of ferritin. NCOA4 binds to surface arginine residues on FTH1 through its C-terminal domain, forming a complex that is subsequently engulfed by autophagosomes in a process facilitated by LC3, a key autophagy-related protein. The resulting autophagosomes then fuse with lysosomes to form autophagolysosomes, where ferritin is degraded within the acidic lysosomal environment, releasing bioavailable Fe²^+^ (116). The abundance of NCOA4 directly determines ferritinophagy flux, and its expression is influenced by intracellular iron levels, autophagic activity, lysosomal function, and hypoxic conditions. Reduction of NCOA4 expression limits ferritin degradation and suppresses ferroptosis, whereas NCOA4 overexpression excessively accelerates ferritinophagy, increases intracellular free iron, and triggers ferroptotic cell death. In addition, pharmacological or genetic inhibition of autophagy indirectly blocks NCOA4-mediated ferritin autophagy, thereby reducing ferroptosis initiation (117–119). In the acidic environment of GA-induced inflammation, ASIC1α activation leads to intracellular Ca²^+^ overload. Elevated Ca²^+^ levels trigger liquid–liquid phase separation of the FIP200 complex, resulting in the formation of protein aggregates that interact with endoplasmic reticulum (ER) membrane proteins, including VAPs and ATLs. This interaction induces calcium transients along the ER surface, facilitating autophagosome formation. Subsequently, the interaction between NCOA4 and FTH1 initiates ferritinophagy. Experimental studies show that acid exposure suppresses ASIC1α activity and reduces autophagic flux, whereas enhanced autophagy promotes ferritin degradation. At pH values ≤ 5.5, Fe²^+^ dissociates more readily from ferritin, and the resulting accumulation of free Fe²^+^ drives ferroptotic cell death and amplifies inflammatory responses (120–122). These findings suggest that ASIC1α contributes to cellular ferroptosis by regulating the NCOA4/FTH1 axis, thereby aggravating the pathological progression of GA. Collectively, ferroptosis promotes GA development by shaping oxidative stress and inflammatory signaling, providing new insights for understanding disease mechanisms and identifying potential therapeutic targets (**Figure 3**).

## 4 Summary and outlook

GA is a common metabolic rheumatic disorder, and its progression is closely linked to joint destruction as well as a range of multisystem complications. Current therapeutic strategies remain limited by suboptimal efficacy and notable adverse effects, highlighting the need for new mechanistic insights and targeted interventions. Ferroptosis, a recently characterized iron-dependent form of programmed cell death driven by lipid peroxidation, has been implicated in the pathogenesis of numerous inflammatory diseases. Its defining features include disturbances in iron metabolism, impaired GPX4 activity, and accumulation of lipid peroxides. Its pivotal role in the pathogenesis of GA is closely associated with the abnormal activation of ferroptosis in specific cells, including macrophages, neutrophils, and synovial fibroblasts. Accumulating evidence demonstrates a strong association between iron dysregulation and the development of GA. Clinical studies indicate that serum ferritin and transferrin levels correlate positively with hyperuricemia and GA risk. Iron overload intensifies joint inflammation by enhancing the production of ROS and activating the NLRP3 inflammasome. In addition, ferroptosis may contribute to tophus formation by modulating xanthine oxidase activity and impairing the clearance of MSU crystals. It can also induce synovial and chondrocyte dysfunction, thereby promoting bone erosion and accelerating joint destruction. Emerging pathways, including the FPN1/ROS–ASIC1α axis and the ASIC1α–NCOA4/FTH1 regulatory network, may represent critical mechanistic drivers of GA progression. These insights deepen our understanding of GA pathology and offer promising avenues for the development of novel therapeutic strategies.

Although current research provides preliminary evidence linking ferroptosis to the pathogenesis of GA, several limitations exist. First, the specific role and regulatory mechanisms of ferroptosis at different stages of GA, including the acute flare phase, the chronic intercritical phase, and the tophus formation stage, remain unclear due to a lack of dynamic, stage-specific studies. Second, most available findings are based on cell experiments and animal models, while clinical studies involve small sample sizes. Therefore, the diagnostic and prognostic value of ferroptosis-related biomarkers such as MDA, GSH, and GPX4 activity has not been validated in large patient cohorts. Third, the complex cross-regulatory interactions between ferroptosis and classical inflammatory pathways in GA, such as NF-κB and NLRP3, are not fully understood, and the upstream and downstream relationships among key molecular components require further investigation. Lastly, therapeutic studies targeting ferroptosis have largely focused on traditional approaches such as iron chelators and antioxidants, while the development of new molecularly targeted agents that precisely modulate ferroptosis pathways is still at an early exploratory stage.

Future research on GA and ferroptosis should progress in several key directions. First, a deeper understanding of the stage-specific mechanisms of ferroptosis throughout the GA disease course is needed. Advanced technologies such as single-cell sequencing, spatial transcriptomics, and metabolomics can help delineate the regulatory networks of ferroptosis across different cellular populations, including synoviocytes, chondrocytes, and macrophages. Second, larger clinical cohorts are required to establish standardized detection criteria for ferroptosis-related biomarkers and to evaluate their clinical value in early diagnosis, disease monitoring, and prognostic prediction of GA. Third, further investigation into the interactions between ferroptosis and classical inflammatory pathways, particularly the NLRP3 inflammasome and NF-κB, is essential. This includes deciphering the regulatory roles of key molecules such as ASIC1α, NCOA4, and FPN1 to facilitate the development of multi-target therapeutic strategies. Fourth, intensified efforts should be directed toward the development of new targeted therapeutics, including selective GPX4 activators and ferroptosis inhibitors, with rigorous validation of their efficacy and safety in both *in vitro* and *in vivo* models. Parallel research should also explore how active constituents of traditional Chinese medicine influence ferroptotic pathways. Fifth, integrated therapeutic approaches that simultaneously regulate iron homeostasis and uric acid metabolism should be prioritized, as dual-action strategies may more effectively suppress ferroptosis while reducing uric acid burden. Overall, such approaches hold promise for overcoming current treatment limitations and improving clinical outcomes for GA patients. Additionally, epidemiological and nutritional studies suggest that purine-rich meats, organ meats, and seafood more readily trigger GA flares than purine-rich vegetables. This difference may be closely related to the iron content abundant in animal-based foods and their unique metabolic characteristics. Specifically, iron in animal-derived foods is predominantly heme iron, which exhibits significantly higher bioavailability than plant-based non-heme iron. It is efficiently absorbed through direct binding to intestinal carrier proteins, released as ferrous iron after heme oxygenase cleavage, and enters the bloodstream via FPN1 (123, 124). In contrast, non-heme iron absorption requires prior conversion to ferrous iron and is readily inhibited by dietary components. Additionally, animal-derived foods regulate heme iron release and absorption by altering gut microbiota composition and metabolic products, thereby enhancing iron reserves (125). Excess iron increases gout risk by promoting reactive oxygen species production, activating inflammatory pathways like NF-κB, and exacerbating urate crystal-induced inflammation (126). This mechanism elucidates the differential gout induction between the two high-purine food categories. Further investigation into this relationship may provide valuable dietary guidance for GA patients and support early preventive strategies for individuals at high risk, such as those with hyperuricemia. These insights could help reduce GA incidence and offer meaningful benefits for both clinical practice and public health.
