# Correction: Ferroptosis in gouty arthritis: a potential therapeutic strategy

**DOI:** 10.3389/fimmu.2026.1811266

**Published:** 2026-02-23

**Authors:** Heguo Yan, Niqin Xiao, Bo Yang, Jian Zhang, Yundong Xu, Bingbing Chen, Sanjin Zeng, He Qian, Shengyi Zhao, Rong Wang, Zhaohu Xie, Zhaofu Li, Jing Xie

**Affiliations:** 1 Yunnan University of Chinese Medicine, Kunming, Yunnan, China; 2 Zhaotong Hospital of Traditional Chinese Medicine, Zhaotong, Yunnan, China

**Keywords:** cell death, ferroptosis, gouty arthritis, inflammatory response, targeted therapy

Authors “Bingbing Chen, Sanjin Zeng, He Qian, Shengyi Zhao, Rong Wang, Zhaohu Xie and Jing Xie” were erroneously assigned to affiliation “3 School of Basic Medical Sciences, Yunnan University of Chinese Medicine, Kunming, Yunnan, China”. This affiliation has now been removed for author “Bingbing Chen, Sanjin Zeng, He Qian, Shengyi Zhao, Rong Wang, Zhaohu Xie and Jing Xie” and the above authors’ affiliation as been changed to “1 Yunnan University of Chinese Medicine, Kunming, Yunnan, China”.

Author “Zhaofu Li” was erroneously assigned to affiliation “4 Department of Science and Technology, Yunnan University of Chinese Medicine, Kunming, Yunnan, China”. This affiliation has now been removed for author “Zhaofu Li”, and his affiliation has been changed to “1 Yunnan University of Chinese Medicine, Kunming, Yunnan, China”.

Affiliation “1 Yunnan University of Chinese Medicine, Kunming, Yunnan, China” was erroneously given as “1 The First Clinical Medical College of Yunnan University of Chinese Medicine, Kunming, Yunnan, China”.

The original version of this article has been updated.

An incorrect **Funding** statement was provided. The correct **Funding** statement reads:

“The author(s) declared that financial support was received for this work and/or its publication. This work was funded by the National Natural Science Foundation of China (Grant No. 82260925); the Yunnan Provincial High-level Scientific and Technological Talents and Innovative Teams Selection Special Project (Grant No. 202305AS350007); the Yunnan Provincial High-Level Traditional Chinese Medicine Talent Cultivation Program for Successor Talent in TCM Disciplines (Yun Cai She [2024] No. 103); the Yunnan Provincial Department of Science and Technology Science and Technology Plan Projects (202101AZ070001-140, 202101AZ070001-312, 202401AZ070001- 091, 202401AU070065); the Open Project of the Yunnan Provincial Key Laboratory of Dai and Yi Medicine (2024SS24010); and the Open Research Fund Program of Yunnan Key Laboratory of Integrated Traditional Chinese and Western Medicine for Chronic Disease in Prevention and Treatment (YPKLS2024-006, 2019DG016).”

The original version of this article has been updated.

